# Idiopathic common carotid artery laceration: A case report

**DOI:** 10.1002/ccr3.6551

**Published:** 2022-11-12

**Authors:** Natsumi Suzuki, Mitsuhiko Katoh, Kento Koda, Masakazu Kuriyama, Gentaro Nagano, Kazuo Yasuhara

**Affiliations:** ^1^ Department of Otolaryngology and Head and Neck Surgery Takeda General Hospital Fukushima Japan; ^2^ Department of Otolaryngology and Head and Neck Surgery The University of Tokyo Tokyo Japan

**Keywords:** carotid artery laceration, carotid blowout syndrome, computed tomography, ultrasonography

## Abstract

Carotid artery injury is a rare disease often caused by neck trauma or irradiation of head and neck cancer. It is not easy to diagnose quickly without these backgrounds. Herein, we report a case of a 52‐year‐old man with no history of trauma or irradiation whose carotid artery was found to be injured. It was suggested that patients without any other disease but hypertension could have carotid artery injury. Additionally, it was challenging to detect extravasation from the carotid artery by computed tomography scan and ultrasonography in the emergency department. We should consider the possibility of carotid artery injury when examining patients with sudden neck swelling, even without any history of trauma or irradiation.

## Graphical abstract

Pay close attention to the cervical swelling, which can progress in a few hours. An ultrasound around the carotid artery and a contrast‐enhanced CT scan of the neck should be performed as soon as possible.

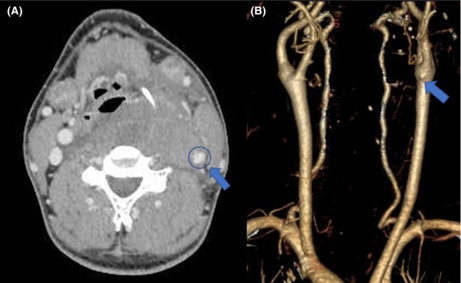

## 1 INTRODUCTION

Carotid artery injury occurs in patients with neck trauma, head and neck cancer, and vasculitis.^1^, ^2^, ^3^ It is challenging to diagnose and treat rapidly because it is a rare disease, and the appropriate choice of treatment approach, whether endovascular or surgical, is not established. We report a case of carotid artery laceration in a patient with no specific history, except for hypertension, diagnosed only after the surgery to investigate the cause of neck swelling.

## 2 CASE PRESENTATION

A 52‐year‐old man with a history of hypertension and smoking (32 pack‐years) presented to our emergency department with sudden neck pain and swelling for 3 h before consultation, without any apparent trigger. He had no history of neck surgery, puncture, trauma, or irradiation. His body temperature, blood pressure, heart rate, and SpO_2_ were 36.2°C, 177/111 mmHg, 75 beats/min, and 99%, respectively. Physical examination revealed swelling, gradually getting harder, on the left side of the neck.

**FIGURE 1:**
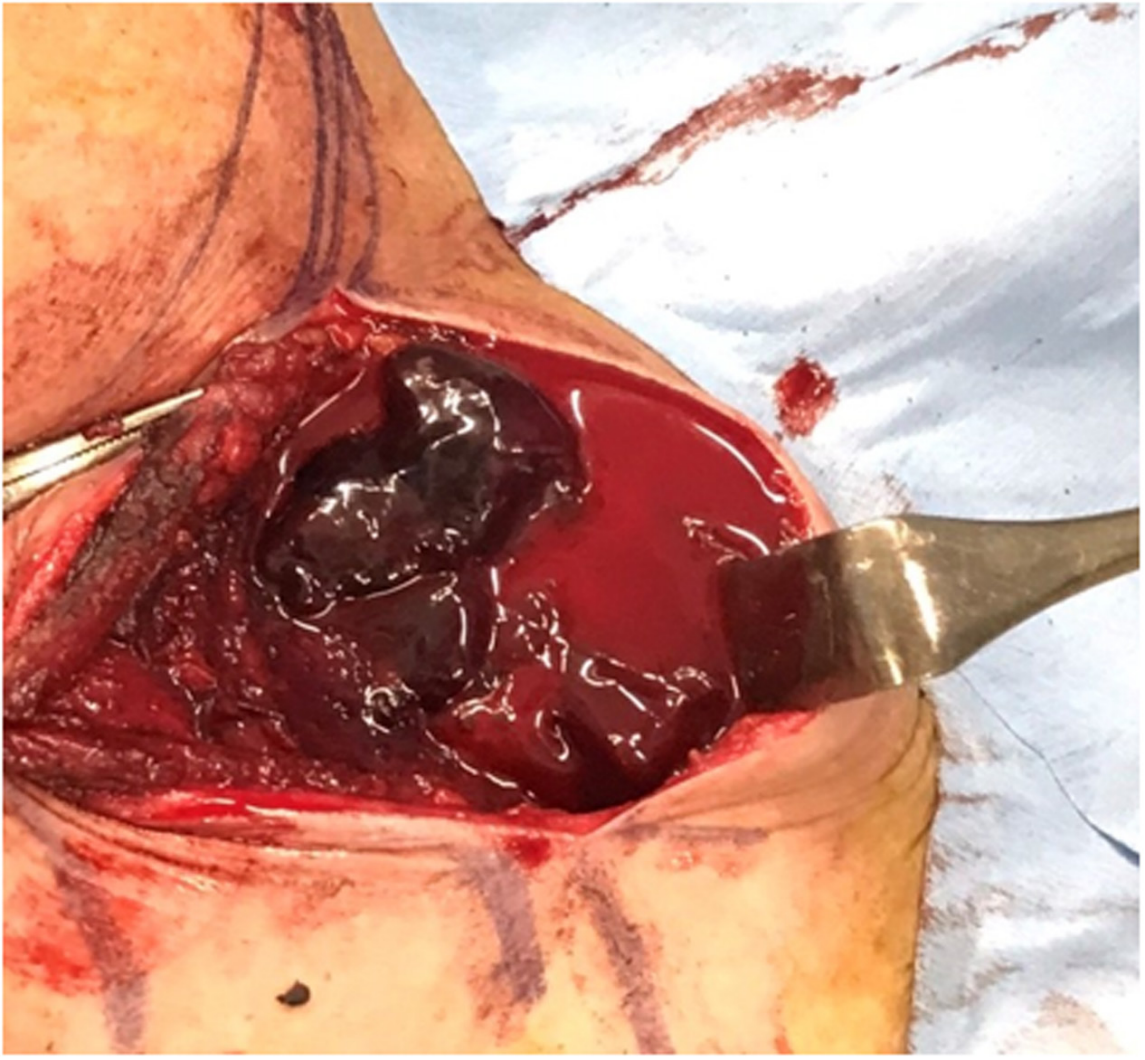
A skin incision made over the sternocleidomastoid muscle to easily locate and reach the hematoma cavity

**FIGURE 2:**
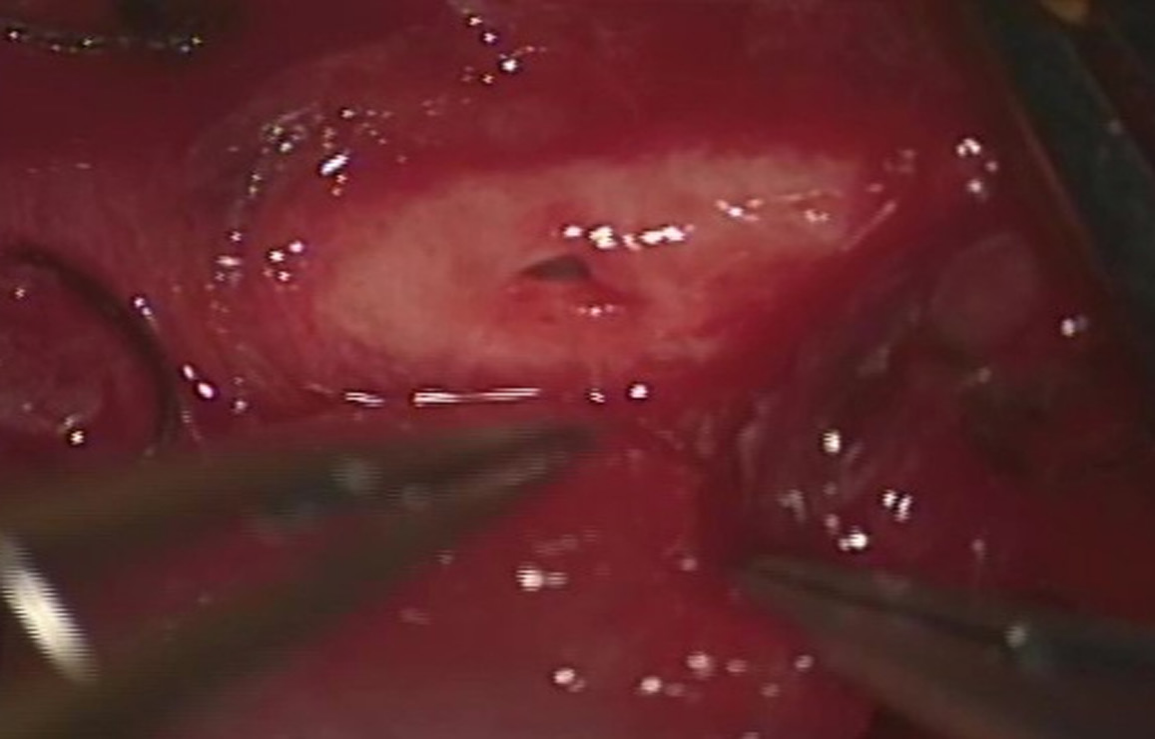
When blood flow was temporarily blocked with a vascular tape, a blunt injury of approximately 3 mm was observed on the medial side of the carotid artery

**FIGURE 3:**
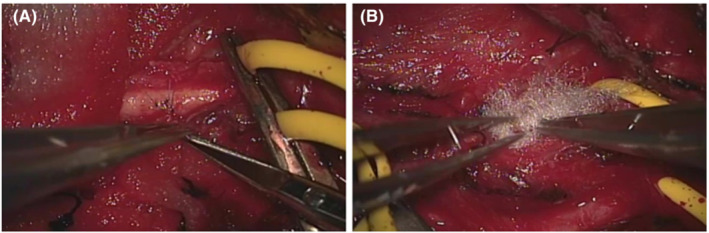
(A) The laceration was closed under microscopic guidance using an 8–0 proline suture in the shortaxis direction. (B) The surrounding area was covered with Surgicel cotton

A laryngeal fiberscope showed slowly progressive laryngeal edema without a mass or bleeding. Contrast‐enhanced computed tomography (CT) showed a widespread low‐absorption area without contrast effect in the left cervical region, but no extravasation was observed in both arterial and venous phases. Ultrasonography showed uniform hypoechoic areas without blood flow around the cervical vessels and the thyroid gland. Based on these findings, we could not diagnose the source of bleeding, but we considered that the neck swelling was caused by a hematoma in the left side of the neck. We suspected bleeding from the parathyroid gland. To find the bleeding point and stop the bleeding, we performed surgery under general anesthesia to remove the hematoma. A skin incision was made over the sternocleidomastoid muscle to reach the hematoma cavity quickly (Figure 1). Blood spurted from the carotid artery when most of the hematoma was removed. There was a blunt laceration of approximately 3 mm on the medial side of the common carotid artery (Figure 2). We blocked the left carotid artery temporarily and sutured the laceration using 8‐0 absorbable sutures under microscopy (circulation was blocked for 31 min). After that, we covered the sutured artery with SURGICEL® FIBRILLAR™ (Figure 3A,B). Tracheostomy was performed to secure the airway because it was possible that the laryngeal edema could have deteriorated if postoperative bleeding occurred. The intraoperative bleeding volume was 537 ml, and postoperative hemoglobin levels remained stable; therefore, blood transfusion was not performed. There were no cerebral or neurological complications, and the rehabilitation course was favorable. Surgical tracheostomy closure was performed on postoperative day 15, and the patient was discharged on postoperative day 18.

## 3 DISCUSSION AND CONCLUSIONS

We presented a case of carotid artery laceration without trauma, irradiation, or vasculitis, which is difficult to diagnose in the emergency department. Our case suggested two clinically essential points. (1) We should consider the possibility of common carotid artery laceration in patients with sudden neck swelling. (2) In the emergency department, it can be challenging to find bleeding points, even if CT and ultrasonography are performed.

Carotid artery laceration is a rare but catastrophic disease; in the worst case, it leads to death. It has been reported that neck trauma, treatments of head and neck cancer, and vasculitis often cause carotid artery rupture; it is well known as carotid blowout syndrome (CBS) in patients with head and neck cancer.^1^, ^2^

Carotid blowout syndrome is a rare complication of surgical procedures and (chemo)radiotherapy for head and neck cancer. It is believed that radiation‐induced free radicals cause occlusion of the adventitial vasa vasorum, fibrosis, premature atherosclerosis, and weakening of the arterial wall. These vascular changes lead to carotid artery rupture.^1^, ^2^ Carotid artery aneurysm, and its rupture in patients with vasculitis are rare; only a few reports showed carotid artery rupture with vasculitis such as Takayasu arteritis.^3^

In the present case, the patient did not have a history of neck trauma or head and neck cancer. In addition, he did not have a fever at the time of presentation, and no symptoms such as headache, dizziness, fatigue, skin rash, or joint swelling or pain; he was not suspected to have vasculitis. Fibrosis or aneurysm was not observed in the surgery. Therefore, it was suggested that there was no apparent cause of carotid injury; we concluded it was idiopathic carotid laceration, like idiopathic carotid dissection.

Idiopathic carotid dissection, a significant cause of cerebral infarction at younger ages, is known to occur suddenly.^4^ A previous report showed that 27% of patients with idiopathic carotid dissection had a history of hypertension, 41% of patients had a history of smoking, and the mean age was 43.6 years old.^5^ Another report showed that carotid dissection had no identifiable cause in 70% of patients.^6^ In this case, a middle‐aged patient without HNC and vasculitis but with hypertension developed carotid injury. Although we did not find evidence of an aneurysm or dissection in surgery, our case was similar to cases of idiopathic carotid dissection in the onset pattern, onset age, and a history of hypertension. Therefore, we speculated that the cause of the idiopathic carotid laceration was similar to that of idiopathic carotid dissection. Unfortunately, we did not obtain the tissue around the laceration and we could not analyze the pathogenesis through the specimen. To prevent the recurrence of carotid rupture, oral administration of amlodipine was started after the operation, and the patient's systolic blood pressure was maintained between 120 and 140 mmHg during hospitalization. This range was slightly higher than normal, but it was essential to prevent cerebral ischemia after carotid artery repair.

We diagnosed carotid artery laceration during surgery; however, no extravasation was detected by CT scan and ultrasonography in the emergency department. We initially suspected bleeding from parathyroid adenoma.^7^

**FIGURE 4:**
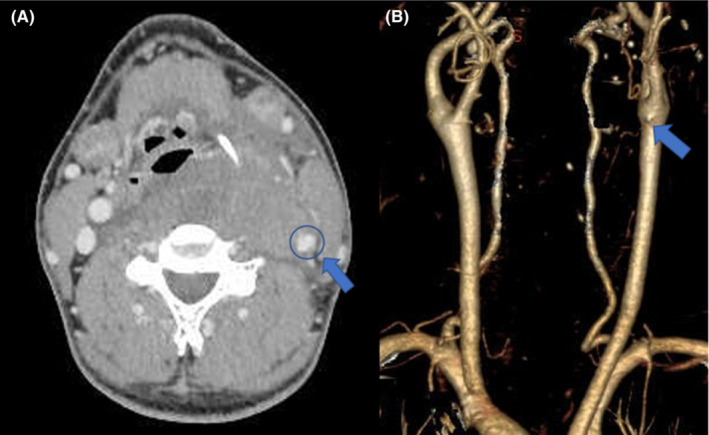
(A) A small extravasation that appeared to be a hemorrhage point was observed in one slice of the computed tomography image. (B) Postoperative three‐dimensional images also revealed that the same site was the source of the idiopathic hemorrhage

To detect these bleedings, angiography seemed very useful^2^; unfortunately, we did not have access to perform angiography in our hospital. Endovascular treatments for vascular injury, including stenting and embolization, were believed to be effective in particular cases.^2^ Still, our patient needed to have his airway secured because impaired blood flow due to compression of the internal jugular vein by the progressive hematoma could have caused edema, leading to airway obstruction. Also, because of airway obstruction, we did not consult another hospital to perform angiography. After the operation, we reviewed the CT images and found small extravasation of contrast in one image slice(Figure 4A,B). We initially suspected bleeding from the parathyroid gland, which has been reported in many cases, but we did not manage to make the correct diagnosis.

A large hematoma, like in this case, induces infection by itself, and it was essential to remove it surgically. In this regard, the surgical approach to the hematoma was necessary in this case, even if the endovascular approach was accessible. Additionally, tracheotomy was important to secure the airway. It can be performed in the same operative field as the carotid artery repair and has the advantage of allowing management in the awake state and shortening intubation time.

This case suggests that we had better suspect carotid rupture when examining the cause of sudden neck swelling, even though the patient does not have any history of HNC or vasculitis. In addition, it was challenging to detect bleeding from the carotid laceration by CT and ultrasonography scan in the emergency department, and screening along the carotid artery should be carefully done.

## AUTHOR CONTRIBUTIONS

Natsumi Suzuki wrote the initial draft. Mitsuhiko Katoh was involved in patient care, wrote and edited the draft, and equally supervised the study. Kento Koda was involved in patient care and follow‐up and conceived and supervised the study. Masakazu Kuriyama, Gentaro Nagano, and Kazuo Yasuhara were involved in patient care and critically reviewed the manuscript.

## CONFLICT OF INTEREST

The authors declare that they have no competing interests.

## CONSENT

Written informed consent was obtained from the patient for the publication of this case report and the accompanying images.

## Data Availability

The data that support the findings of this study are available on request from the corresponding author. The data are not publicly available due to privacy reasons.
